# Sexually transmitted infections after bereavement – a population-based cohort study

**DOI:** 10.1186/s12879-016-1705-x

**Published:** 2016-08-15

**Authors:** Emily Bond, Donghao Lu, Eva Herweijer, Karin Sundström, Unnur Valdimarsdóttir, Katja Fall, Lisen Arnheim-Dahlström, Pär Sparén, Fang Fang

**Affiliations:** 1 Department of Medical Epidemiology and Biostatistics, Karolinska Institutet, Nobels väg 12A, Box 281, Stockholm, 17177 Sweden; 2 Department of Laboratory Medicine, Karolinska Institutet, H5, Division of Pathology, Karolinska Universitetssjukhuset Huddinge, Stockholm, 14186 Sweden; 3 Center of Public Health Sciences, Faculty of Medicine, University of Iceland, Stapi v/Hringbraut, 101 Reykjavik, Iceland; 4 Department of Epidemiology, Harvard School of Public Health, 677 Huntington Avenue, Boston, MA 02115 USA; 5 Clinical Epidemiology and Biostatistics, School of Medical Sciences, Örebro University, Örebro, Sweden

**Keywords:** Sexually transmitted infections, Bereavement, Psychological stress, Salpingitis, Condyloma, HPV vaccination

## Abstract

**Background:**

Loss of a loved one has consistently been associated with various health risks. Little is however known about its relation to sexually transmitted infections (STIs).

**Methods:**

We conducted a population-based cohort study during 1987–2012 using the Swedish Multi-Generation Register, including 3,002,209 women aged 10-44 years. Bereavement was defined as death of a child, parent, sibling or spouse (*N* = 979,579, 33 %). STIs were defined as hospital visits with an STI as main or secondary diagnosis. Poisson regression and negative binomial regression were used to estimate incidence rate ratios (IRRs) and 95 % confidence intervals (CIs) of STIs, comparing incidence rates of women who had experienced loss to those who had not.

**Results:**

Bereaved women were at significantly higher risk of nearly all STIs studied. The relative risk of any STI was highest during the first year after loss (IRR: 1.45, 95 % CI: 1.27–1.65) and predominantly among women with subsequent onset of psychiatric disorders after bereavement (IRR: 2.61, 95 % CI: 2.00–3.34). Notably, a consistent excess risk, persisting for over five years, was observed for acute salpingitis (IRR: 1.28, 95 % CI: 1.13–1.44), a severe complication of bacterial STIs.

**Conclusion:**

These data suggest that women who have experienced bereavement are at increased risk of STIs.

**Electronic supplementary material:**

The online version of this article (doi:10.1186/s12879-016-1705-x) contains supplementary material, which is available to authorized users.

## Background

That psychological stress has immune modulating effects is suggested by extensive experimental evidence from both animal [1] and human [2] studies. Experimental studies have further suggested that psychological stress increases susceptibility to viral infections [3]. On the contrary, epidemiological evidence for the potential impact of psychological stress, including various life stressors, on the susceptibility to infections remains weak [4]. However, multiple meta-analyses have shown that psychological stress might facilitate the recurrence of herpes simplex virus infection [5] and the progression of human immunodeficiency virus (HIV) infection [6], lending evidence to theories of psychological stress impairing our ability to control infection.

Sexually transmitted infections, including infections of human papillomavirus (HPV), are major, or even necessary, causes for many infection-related cancers [7, 8]. While the literature on the role of psychological stress in cancer development remains largely inconclusive [9, 10], three recent large population-based studies have suggested a higher risk of cervical cancer, and other HPV related cancers, among bereaved women [11–13]. Although the role of oncologic infections of HPV has been highlighted [13], little is known about whether the underlying mechanism linking bereavement to cervical cancer can be attributed to behavioral or biological changes.

To this end, in the present study, we aimed to complement these previous studies by elucidating whether there is an increased risk of sexually transmitted infections (STIs) after bereavement.

## Methods

### Study population

**Fig. 1:**
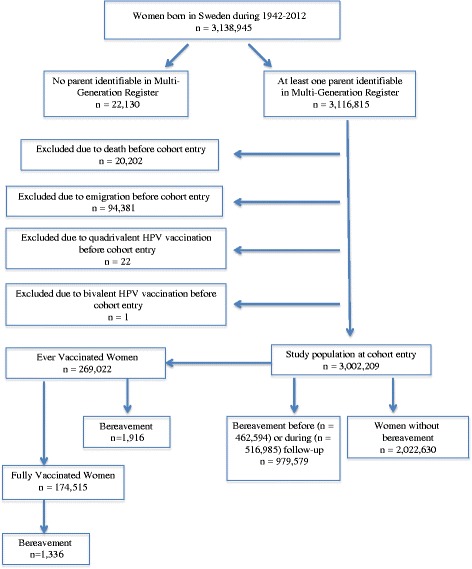
Study population. The study population included all women born in Sweden who were at the age of 10–44 years during 1987–2012, and had at least one parent identifiable in the Multi-Generation Register. Women who received HPV vaccination during the study period were analyzed separately

All women born in Sweden, who were at the age of 10–44 years during 1987–2012, who had at least one parent registered in the Swedish Multi-Generation Register (MGR), were included in the present study (*N* = 3,002,209) (Fig. 1). The MGR contains largely complete familial linkages for all Swedish residents born since 1932 onward [14].

### Follow-up

All women were individually followed from January 1^st^ 1987 or their tenth birthday, whichever came later, to an STI diagnosis, death, emigration, 45^th^ birthday, HPV vaccination, or December 31^st^ 2012, whichever came first, through cross-linkages to the Swedish Patient Register (for STI diagnosis), Cause of Death Register, Migration Register, as well as the Swedish Vaccination Register and the Prescribed Drug Register (collectively for HPV vaccination). Women receiving an HPV vaccination were further studied in a separate analysis.

The Patient Register collects hospital discharge records in Sweden since 1964/1965, and has a complete coverage for the entire country since 1987 [15]. Hospital-based outpatient specialist visits have been added in this register since 2001 with >80 % coverage of the entire country. The Patient Register does however not include visits to general practitioners. The 9^th^ Swedish revision of the International Classification of Diseases (ICD-9) codes was used in this register between 1987 and 1996 and the 10^th^ revision thereafter.

As previously described [16, 17], date for HPV vaccination was first retrieved from the Swedish Vaccination Register, and complemented by prescription date of HPV vaccination (Anatomical Therapeutic Chemical (ATC) codes J07BM01 and J07BM02) according to the Prescribed Drug Register when needed. The Prescribed Drug Register records all prescribed drugs from all pharmacies in Sweden since July 2005 [18].

Women without bereavement contributed all person-time to the unexposed group, whereas the exposed women contributed person-time to the unexposed group before the bereavement and to the exposed group thereafter. If a woman had bereavement before entry to the cohort, she contributed person-time only to the exposed group. If a woman lost more than one family member, the first bereavement was counted.

### Bereavement

We used the MGR to identify the first-degree relatives of all index women in our study population, including children, parents, and siblings. Spouses were defined through a registered common biological or adopted child in the MGR. We then linked all the family members to the Cause of Death Register and identified any death of these family members from January 1^st^ 1961 until the end of study (December 31^st^ 2012). Bereavement was first classified based on the relationship of the index women to the lost kin (“type of bereavement”: loss of a child, spouse, parent, or sibling), and then categorized by cause of death (“cause of bereavement”: self-harm, other injuries, cancer, or non-cancer diseases).

### Sexually transmitted diseases and related outcomes

STIs were defined through hospital visits where STIs were recorded either as the main or a secondary diagnosis. The STIs studied in the present study included condyloma (i.e. Condyloma acuminatum), gonorrhea, chlamydia, syphilis, and genital herpes simplex (GHS) (Additional file 1: Table S1). Any STI was defined as the composite of the above STIs. The first STI diagnosis during follow-up was used as the outcome of interest for both the exposed and unexposed groups. As a result, bereaved women might have one STI diagnosis before bereavement and another STI diagnosis after bereavement. Concurrence of multiple STIs at the same hospital visit was possible and in such case, the specific STI recorded as the main diagnosis of the visit was used as the outcome of interest. Since we had complete data only on inpatient hospital records for the entire study period, we used the first inpatient hospital contact for STIs as the primary outcome. To assess the representativeness of our findings, we used any hospital contact (both inpatient and outpatient) as secondary outcome for the period of 2001–2010. As another secondary outcome, we complemented the definition of condyloma by using information from the Prescribed Drug Register as previously described [16, 17] for the period of July 2005 to December 2012. Briefly, prescriptions for the condyloma treatments, Aldara and Podophylotoxin (under age of 45 [16]), were identified with ATC codes D06BB10 and D06BB04 and used as alternative definitions of condyloma diagnosis.

Acute salpingitis, inflammation of the fallopian tubes, is not an STI by definition, but it largely shares risk factors with STIs [19]. Furthermore, it has been shown that the rates of acute salpingitis follow chlamydia epidemics very closely [20]. Although some of the STIs studied in the present study may also be diagnosed and treated in clinics outside hospitals (e.g., with general practitioners or youth clinics) and are therefore incompletely covered by the Patient Register, acute salpingitis is more consistently diagnosed and treated within hospital-based clinics.

We additionally studied infections of human immunodeficiency virus-1 (HIV) and hepatitis B (HBV) as STIs with other potential routes of transmission (including intravenous drug use) in addition to high risk sexual behavior.

### Separate analysis of HPV vaccinated women

To better understand the potential impact of immune modulation on the studied associations, we conducted a separate analysis among women vaccinated with quadrivalent HPV vaccine for the period of June 2006 to December 2012. Our hypothesis was that the severe psychological stress induced by bereavement might compromise the host immunity in terms of its ability to mount an adequate response to vaccination – an exposure to HPV antigens unrelated to sexual behavior – and therefore result in a suboptimal vaccination efficacy. The quadrivalent HPV vaccine protects against HPV types 6, 11, 16 and 18 which cause about 90 % of condyloma [21]. In total, we identified 269,022 women who received at least one dose of quadrivalent vaccine (“ever vaccinated women”) and 174,515 women who received all three doses of quadrivalent vaccine (“fully vaccinated women”) (Fig. 1). For this analysis, ever vaccinated women were followed from the date of first dose and fully vaccinated women from the date of third dose.

### Statistical analysis

We first calculated the unadjusted incidence rates (i.e., number of STIs divided by accumulated person-years) among women with and without bereavement separately. We then used log-linear Poisson regression models to estimate the incidence rate ratios (IRRs) and 95 % confidence intervals (CIs) of STIs of the bereaved women, compared to women without bereavement. If the Poisson model was found to be over-dispersed, negative binomial regression was used instead. All models were adjusted for age at follow-up (10-18 and 19-44 years for the analysis of vaccinated women; and 10–12, 13–15, 16–18, 19–21, 22–25, 26–29, 30–34, 35–39, and 40–44 years for the rest of the analyses), parental educational level and calendar period at follow-up (5-year groups). Information on parental educational level was obtained from linking the parents of the participants to the Swedish Education Register. Since we studied relatively young women (10–44 years), parental educational level was used as a proxy for the index woman’s socioeconomic status. Three educational levels were defined, including low (<9 years or missing), middle (9 years plus 2-3 years of high school), and high (university or doctoral studies). These analyses were performed for both the primary outcome (i.e., inpatient hospital contact for STIs for the period of 1987–2012) and secondary outcome (i.e., any hospital contact for STIs for the period of 2001–2012; and any hospital contact plus prescription claim for condyloma for the period of 2005–2012).

To alleviate potential concern of unmeasured confounders, in a sensitivity analysis, we restricted the analyses to women with bereavement and compared the risk of STIs during the time period after bereavement with the risk during the time period before bereavement.

We further investigated the impact of type and cause of bereavement as well as time since bereavement (≤1 year, 2–4 years, or ≥5 years) in association with the inpatient hospital contact of any STI, acute salpingitis, and HIV or HBV (HIV/HBV). We further stratified the analyses of any STI, acute salpingitis, and HIV/HBV by age at follow-up (10–18, 19–29 and 30–44 years), calendar period of follow-up (1987–2000 and 2001–2012), and parental educational level.

Since mental illness is a known risk factor for high risk sexual behavior and STIs [22, 23], we ascertained the history of psychiatric disorders for all women according to the Patient Register (including both inpatient and outpatient hospital contact as well as main and secondary diagnoses for each visit), starting from 1981 to the end of 2012 (ICD-8/9 codes 290–319 during 1981–1996; and ICD-10 codes F00-F99 since 1997). Patients were classified as having preexisting psychiatric disorders during the entire follow-up if they had a diagnosis for any psychiatric disease before entry to the cohort or from the date of such diagnosis if after entry to the cohort. To examine whether preexisting psychiatric disorders might modify the association of bereavement with STIs, we first stratified the analyses by the history of psychiatric disorders. To examine whether psychiatric disorders might mediate the association, we additionally classified the bereaved women as with or without newly onset psychiatric disorders after bereavement, and compared their risk of STIs with women without bereavement. Bereaved women with preexisting psychiatric disorders were excluded from this analysis. Since HIV/HBV infections are highly related to substance abuse, we conducted specifically similar analyses for substance abuse (ICD-8, 304; ICD-9, 304 and 305X; ICD-10, F11-F16 and F18-F19).

Provided with a smaller number of participants, among HPV-vaccinated women, ever vaccinated or fully vaccinated, we only calculated the IRRs of condyloma and any STI. This analysis was performed only for secondary STI outcomes, i.e., both inpatient and outpatient hospital visits for any STI, and hospital visits plus prescribed drugs for condyloma. In organized vaccination programs, testing for prevalent HPV infection at the time of vaccination is not performed, and HPV status at vaccination is therefore unknown. Since an incubation period of three months has previously been observed for condyloma [17], in a sensitivity analysis, we excluded the first three months of follow-up.

Statistical analyses were conducted using SAS version 9.3 (SAS Institute, Inc.).

## Results

The study included 3,002,209 women with an average follow-up of 14.4 years (Fig. 1). 979,579 of the entire cohort and 1916 of the vaccinated women experienced bereavement before or during follow-up.

Compared to the unexposed group, the exposed group had older age at cohort entry, lower parental education level, and less preexisting psychiatric diseases (Additional file 1: Table S2).

Overall, bereaved women had a higher risk of any STI, using either the primary (inpatient hospital visit; IRR: 1.16, 95 % CI: 1.08–1.24) or secondary (any hospital visit; IRR: 1.06, 95 % CI: 1.01-1.11) definition (Table 1). Individual STIs with significantly elevated risk among bereaved women included condyloma and chlamydia (Table 1). Bereaved women also had increased risks of acute salpingitis, HIV and HBV infections (Table 1).

**Table 1:** Hospital contact of Sexually Transmitted Infections (STI), comparing bereaved women with non-bereaved women

	Inpatient hospital contact (1987–2012)					Any hospital contact (2001–2012)				
	No bereavement		Bereavement			No bereavement		Bereavement		
	N	Crude IR (100,000 PYs)	N	Crude IR (100,000 PYs)	IRR (95 % CI)	N	Crude IR (100,000 PYs)	N	Crude IR (100,000 PYs)	IRR (95 % CI)
Any STI	5903	17.00	1188	14.50	1.16 (1.08–1.24)	71061	453.00	9309	304.00	1.06 (1.01–1.11) ^*b*^
Condyloma ^*a*^	2833	8.14	700	8.54	1.18 (1.08–1.30)	42652	272.00	5334	174.00	1.06 (1.00–1.12) ^*b*^
Gonorrhea	123	0.35	33	0.40	1.51 (0.97–2.28)	466	2.97	75	2.45	1.49 (1.14–1.92)
Chlamydia	952	2.74	136	1.66	1.23 (1.01–1.48)	10098	64.40	1185	38.60	1.14 (1.04–1.26) ^*b*^
Syphilis	65	0.19	20	0.24	1.17 (0.66–2.01)	72	0.46	16	0.52	1.05 (0.57–1.84)
GHS	1946	5.59	302	3.68	1.06 (0.93–1.21)	17828	114.00	2709	88.30	1.04 (0.99–1.09) ^*b*^
Acute salpingitis	11361	32.60	3540	43.20	1.28 (1.13–1.44)^*b*^	2929	18.70	1000	32.60	1.24 (1.15–1.34)
HIV	120	0.34	96	1.17	2.36 (1.76–3.17)	220	1.40	112	3.65	1.98 (1.53–2.53)
HBV	435	1.25	161	1.96	1.59 (1.30–1.94)	411	2.62	141	4.60	2.20 (1.78–2.71)

Notes:

The first hospital visit concerning an STI diagnosis during the unexposed follow-up and the first hospital visit concerning an STI diagnosis during the exposed follow-up were both counted in all analyses

*CI* confidence interval, *GHS* genital herpes simplex, *HBV* hepatitis B virus, *HIV* human immunodeficiency virus, *IR* incidence rate, *IRR* incidence rate ratio, *N* number, *PYs* person-years, *STI* sexually transmitted infection

IRR was adjusted for attained age (10–12, 13–15, 16–18, 19–21, 22–25, 26–29, 30–34, 35–39, and 40–44), calendar years (5-year group), and parental education levels (low/missing, medium, and high) as a proxy for socioeconomic status

^*a*^ Any hospital contact for condyloma was further complemented by prescription claim

^*b*^ Negative binomial regression was used instead due to over-dispersed Poisson regression

Similar findings were noted when comparing the pre-bereavement to post-bereavement periods of the same women, although the magnitude of the associations was slightly diminished (Table 2).

**Table 2:** Inpatient hospital contact of Sexually Transmitted Infections (STIs), comparing the post-bereavement to the pre-bereavement periods of women with bereavement

	Before bereavement		After bereavement		
	N	Crude IR (1000 PYs)	N	Crude IR (1000 PYs)	IRR (95 % CI)
Any STI	1428	0.26	1188	0.14	1.14 (1.05–1.25)
Condyloma ^*a*^	831	0.15	700	0.09	1.15 (1.03–1.29)
Gonorrhea	21	0.00	33	0.00	2.71 (1.49–5.00)
Chlamydia	194	0.04	136	0.02	1.15 (0.90–1.46)
Syphilis	12	0.00	20	0.00	1.21 (0.55–2.76)
GHS	375	0.07	302	0.04	1.00 (0.84–1.18)
Acute salpingitis	3569	0.66	3540	0.43	1.09 (0.95–1.27)^*b*^
HIV	21	0.00	96	0.01	2.36 (1.46–3.99)
HBV	84	0.02	161	0.02	1.41 (1.06–1.89)

Notes:

The first hospital visit concerning an STI diagnosis before bereavement and the first hospital visit concerning an STI diagnosis after bereavement were both counted in all analyses

*CI* confidence interval, *GHS* genital herpes simplex, *HBV* hepatitis B virus, *HIV* human immunodeficiency virus, *IR* incidence rate, *IRR* incidence rate ratio, *N* number; PYs, person-years

IRR was adjusted for attained age (10–12, 13–15, 16–18, 19–21, 22–25, 26–29, 30–34, 35–39, and 40–44), calendar years (5-year group), and parental education levels (low/missing, medium, and high) as a proxy for socioeconomic status

^*a*^ Any hospital contact for condyloma was further complemented by prescription claim

^*b*^ Negative binomial regression was used instead due to over-dispersed Poisson regression

Bereavement characteristics did not clearly modify the risks of any STI, acute salpingitis, or HIV/HBV infections, although slightly stronger associations with loss of a spouse or sibling, as well as bereavement due to self-harm or other injuries, were suggested for some STIs (Table 3). The strongest associations for any STI (IRR: 1.45, 95 % CI: 1.27–1.65) and acute salpingitis (IRR: 1.49, 95 % CI: 1.25-1.79) were seen during the first year after bereavement (Table 3).

**Table 3:** Inpatient hospital contact of Sexually Transmitted Infections (STIs), by causes, types, and recentness of bereavement

	Any STI		Acute salpingitis		HIV/HBV	
	N	IRR (95 % CI)	N	IRR (95 % CI) ^*a*^	N	IRR (95 % CI)
No bereavement	5903	1.0	11361	1.0	555	1.0
Type of bereavement						
Loss of a child	26	1.05 (0.69–1.51)	119	1.35 (1.03–1.76)	8	2.24 (1.01–4.22)
Loss of a spouse	28	1.81 (1.21–2.57)	94	1.48 (1.11–1.98)	18	6.88 (4.11–10.8)
Loss of a parent	924	1.13 (1.04–1.21)	2779	1.25 (1.10–1.42)	194	1.67 (1.39–2.00)
Loss of a sibling	210	1.24 (1.08–1.42)	548	1.41 (1.21–1.65)	37	1.83 (1.28–2.52)
Cause of bereavement						
Due to self-harm	160	1.16 (0.99–1.35)	510	1.54 (1.31–1.80)	42	2.49 (1.78–3.37)
Due to other injury	92	1.21 (0.98–1.48)	251	1.38 (1.14–1.66)	26	2.75 (1.80–4.00)
Due to cancer	314	1.09 (0.97–1.23)	935	1.18 (1.02–1.36)	59	1.36 (1.02–1.79)
Due to non-cancer diseases	622	1.18 (1.08–1.29)	1844	1.31 (1.15–1.50)	130	1.73 (1.40–2.12)
Time since bereavement						
First year	262	1.45 (1.27–1.65)	699	1.49 (1.25–1.79)	23	1.57 (0.99–2.37)
2–4 years	564	1.08 (0.98–1.18)	1628	1.16 (1.00–1.34)	74	1.70 (1.30–2.19)
≥ 5 years	362	1.07 (0.96–1.20)	1217	1.30 (1.13–1.51)	160	1.85 (1.51–2.25)

Notes:

The first hospital visit concerning an STI diagnosis during the unexposed follow-up and the first hospital visit concerning an STI diagnosis during the exposed follow-up were both counted in all analyses

*CI* confidence interval, *HBV* hepatitis B virus, *HIV* human immunodeficiency virus, *IRR* incidence rate ratio, *N* number, *STIs* sexually transmitted infections

IRR was adjusted for attained age (10–12, 13–15, 16–18, 19–21, 22–25, 26–29, 30–34, 35–39, and 40–44), calendar years (5-year group), and parental education levels (low/missing, medium, and high) as a proxy for socioeconomic status

^*a*^ Negative binomial regression was used due to over-dispersed Poisson regression

The associations were not clearly modified by age at follow-up, calendar period of follow-up or parental educational level, except for a slightly stronger association for acute salpingitis among women at age 10-18 years (Table 4).

**Table 4:** Inpatient hospital contact for Sexually Transmitted Infections (STIs), by age, calendar period, parental education level, and psychiatric history

	Any STI			Acute salpingitis			HIV/HBV		
	No bereavement	Bereavement	IRR (95 % CI)	No bereavement	Bereavement	IRR (95 % CI) ^*a*^	No Bereavement	Bereavement	IRR (95 % CI)
Age at follow-up									
10 to 18 years	1181	71	1.15 (0.90–1.45)	1999	163	1.46 (1.24–1.71)	89	6	1.44 (0.56–3.05)
19 to 29 years	3709	494	1.15 (1.04–1.26)	5785	898	1.18 (1.08–1.28)	243	56	1.98 (1.46–2.65)
30 to 44 years	1013	623	1.18 (1.06–1.31)	3577	2479	1.19 (1.07–1.33)	223	195	1.73 (1.41–2.12)
Calendar period of follow-up									
1987 – 2000	4549	980	1.15 (1.06–1.24)	10291	3101	1.24 (1.08–1.42)	292	148	1.65 (1.32–2.05)
2001 – 2012	1354	208	1.19 (1.02–1.39)	1070	439	1.28 (1.13–1.44)	263	109	1.98 (1.54–2.53)
Parental education level									
Low/ Missing	1208	598	1.18 (1.06–1.31)	3173	2063	1.24 (1.00–1.54)	125	125	1.79 (1.37–2.34)
Middle	3002	428	1.15 (1.03–1.27)	5700	1151	1.32 (1.09–1.59)	260	99	2.03 (1.58–2.59)
High	1693	162	1.17 (0.99–1.38)	2488	326	1.25 (1.02–1.54)	170	33	1.48 (0.99–2.17)
History of psychiatric disorders									
No	5353	1121	1.19 (1.11–1.28)	10595	3318	1.28 (1.13–1.45)	352	174	2.08 (1.70–2.56)
Yes	550	67	0.88 (0.67–1.15)	766	222	1.26 (1.01–1.58)	203	83	1.95 (1.46–2.58)
History of substance abuse									
No	5827	1178	1.18 (1.10–1.26)	11253	3502	1.17 (1.13–1.22)	408	206	2.14 (1.77–2.59)
Yes	76	10	0.78 (0.36–1.51)	108	38	1.14 (0.75–1.69)	147	51	1.36 (0.95–1.92)

Notes:

The first hospital visit concerning an STI diagnosis during the unexposed follow-up and the first hospital visit concerning an STI diagnosis during the exposed follow-up were both counted in all analyses

*CI* confidence interval, *HBV* hepatitis B virus, *HIV* human immunodeficiency virus, *IRR* incidence rate ratio, *N* number, *STIs* sexually transmitted infections

IRR was adjusted for attained age (10–12, 13–15, 16–18, 19–21, 22–25, 26–29, 30–34, 35–39, and 40–44), calendar years (5-year group), and parental education levels (low/missing, medium, and high) as a proxy for socioeconomic status

^*a*^ Negative binomial regression was used due to over-dispersed Poisson regression

Apart from a null association between bereavement and any STI among women with preexisting psychiatric disorders, a positive association was noted for acute salpingitis and HIV/HBV, regardless of history of psychiatric disorders (Table 4). Similar result patterns were also noted for substance abuse (Table 4).

Bereaved women with a subsequent psychiatric disorder after bereavement had further increased risks of any STI, compared to bereaved women without psychiatric disorders after bereavement (Table 5). Similarly, more pronounced associations were noted among women with a subsequent diagnosis of substance abuse (Table 5).

**Table 5:** Inpatient hospital contact of Sexually Transmitted Infections (STIs), by subsequent psychiatric disorders or substance abuse after bereavement

	Any STI		Acute salpingitis		HIV & HBV	
	N	IRR (95 % CI)	N	IRR (95 % CI) ^*a*^	N	IRR (95 % CI)
No bereavement	5903	1.0	11361	1.0	555	1.0
Newly diagnosed psychiatric disorder after bereavement						
No	1047	1.07 (1.00–1.15)	3202	1.21 (1.07–1.37)	113	0.85 (0.68–1.05)
Yes	74	2.95 (2.32–3.69)	116	2.09 (1.65–2.62)	61	12.0 (9.00–15.7)
Newly diagnosed substance abuse after bereavement						
No	1160	1.14 (1.06–1.21)	3472	1.26 (1.11–1.42)	150	1.06 (0.87–1.29)
Yes	18	5.12 (3.10–7.87)	30	3.56 (2.36–5.16)	56	76.5 (56.9–100.93)

Notes:

Bereaved women with preexisting psychiatric disorders were excluded from this analysis

The first hospital visit concerning an STI diagnosis during the unexposed follow-up and the first hospital visit concerning an STI diagnosis during the exposed follow-up were both counted in all analyses

*CI* confidence interval, *HBV* hepatitis B virus, *HIV* human immunodeficiency virus, *IRR* incidence rate ratio, *N* number

IRR was adjusted for attained age (10–12, 13–15, 16–18, 19–21, 22–25, 26–29, 30–34, 35–39, and 40–44), calendar years (5-year group), and parental education levels (low/missing, medium, and high) as a proxy for socioeconomic status

^*a*^ Negative binomial regression was used due to over-dispersed Poisson regression

Positive associations of any STI and condyloma were also suggested when only vaccinated women were analyzed, although not statistically significant (Table 6). Similar results were noted when the first three months of follow-up were excluded in this analysis (Additional file 1: Table S3).

**Table 6:** Hospital contact of Sexually Transmitted Infections (STIs) after bereavement, among HPV vaccinated women

	No bereavement			Bereavement		
	N	Crude IR (1000 PYs)	IRR (95 % CI)	N	Crude IR (1000 PYs)	IRR (95 % CI)
Ever vaccinated						
Condyloma	1101	2.03	1.0	12	3.61	1.32 (0.71–2.23)
Any STI	2566	4.73	1.0	23	6.91	1.05 (0.68–1.55)
Fully vaccinated						
Condyloma	584	1.59	1.0	8	3.60	1.86 (0.85–3.50)
Any STI	1696	4.62	1.0	16	7.19	1.27 (0.74–2.00)

Notes:

The first hospital visit concerning an STI diagnosis during the unexposed follow-up and the first hospital visit concerning an STI diagnosis during the exposed follow-up were both counted in all analyses

*CI* confidence interval, *IR* incidence rate, *IRR* incidence rate ratio, *N* number, *PYs* person-years, *STIs* sexually transmitted infections

IRR was adjusted for attained age (10–18 and 19–44), calendar years (5-year group), and parental education levels (low/missing, medium, and high) as a proxy for socioeconomic status

## Discussion

In this population-based cohort study, we found that bereavement, a severely stressful life event, was associated with increased risks of almost all studied STIs among young and middle aged women. The highest risk elevation was noted soon after bereavement and among women with subsequent psychiatric disorders after bereavement. Bereaved women were also more likely to develop acute salpingitis, a severe complication of bacterial STIs.

Sexual risk behavior has been shown to increase among parentally bereaved youth in some [24] but not all [25] studies. In contrast, an elevated risk of depression and substance abuse has been more consistently demonstrated among bereaved adults [26, 27] as well as parentally bereaved youth [28–30]. Increased sexual risk behavior has further been noted among patients with depression, including for example lower rates of contraceptive use [23, 31, 32]. Similarly, substance abuse has also been shown to correlate with sexual risk behavior [33]. Therefore, a possible link between bereavement and STIs might be posited, through altered sexual risk behaviors, either as a direct result of bereavement or secondary to psychiatric conditions consequent to bereavement. Such a link is supported by our finding that, as opposed to a loss of a child, sibling or parent, a slightly higher risk of any STI, and a considerably higher risk of HIV/HBV infection, was detected among women that had lost a spouse - a loss much more likely to lead to a change of sexual partner.

In the present study we observed a higher risk of STIs among bereaved women, regardless of whether or not they were treated in a hospital for psychiatric disorders, including substance abuse, after bereavement, suggesting that clinically diagnosed psychiatric disorders subsequent to bereavement might not be the only underlying pathways for the link of bereavement with STIs. However, since the risk increase was significantly higher among bereaved women with subsequent psychiatric disorders, these conditions appear to be important mediators of the studied association. Studies addressing specific behavior changes after bereavement are warranted to further elucidate these pathways.

In addition to the possible behavioral changes after bereavement, a direct biological impact of bereavement on immune modulation is also plausible [34, 35]. Little data is however available in this regard in terms of STIs. Animal studies have suggested that stress may increase susceptibility to chlamydia [36], and immunosuppression may increase the risk of chlamydia causing salpingitis [37]. Interestingly, the most consistent risk increase after bereavement noted in our study was for acute salpingitis, a severe complication of bacterial STIs. We also found a tendency toward an increased risk of condyloma among women after bereavement, even after HPV vaccination. This could potentially be explained by altered immune modulation after bereavement, leading to a suboptimal vaccine efficacy, or a higher risk of reactivation of pre-vaccination acquired infections. Taken together, our findings might imply that bereavement does not only impact the risk of primary infections through increased exposure and susceptibility, but also induce immunological changes affecting the clearing of the infection, leading to altered risk for complications of the acquired infections.

In previous studies we found an increased risk of cervical cancer in women who had lost a child during adulthood, as well as among women who had lost a parent during childhood [11, 12], potentially mediated through oncologic infections of HPV [13]. The present study lends further support to the potential pathways linking bereavement and cervical cancer, by demonstrating that the risks of STIs and their complications were higher among bereaved women. Hence, increased risk of primary infection together with impaired clearing of acquired infections, may work hand-in-hand to increase the risk of severe complications of STIs, potentially also including cervical cancer.

It is plausible that similar risk factors might lead to bereavement in early to mid-life and a higher risk of STIs. To address this concern, we adjusted for, and stratified by, several known risk factors for STIs in our analyses, such as age, socioeconomic status, and preexisting psychiatric disorders. Residual confounding could still exist since parental educational level might not be a perfect proxy for the women’s socioeconomic status at early to mid-life. In a sensitivity analysis, we restricted the analysis among bereaved women only and compared the risk of STIs before and after bereavement. Even though this comparison had the inert disadvantage of having younger person-years and therefore a higher risk of STIs in the pre-bereavement period, compared to the post-bereavement period, bereavement was still associated with a higher risk of STIs. Comparing different time periods of the same women would largely argue against the possibility that our findings are solely explained by unmeasured confounding.

Since STIs were mainly retrieved from the Patient Register, not including patient visits to the general practitioners, maternity care clinics, and some youth clinics, our definition of STIs was not sensitive. This concern might be alleviated to some extent provided with the findings of condyloma where we used information from inpatient and outpatient hospital contacts as well as through prescription of condyloma medications – a definition with largely improved sensitivity [38] - and reassuringly found still an increased risk, although of smaller magnitude, after bereavement. In line with the clearer association of bereavement with inpatient care of STIs compared to any hospital care for STIs, the overall weaker association noticed for the comprehensive definition of condyloma compared to inpatient care-alone condyloma was expected. This observation should first argue against a pure explanation of altered healthcare seeking behavior among bereaved women leading to an increased detection of STIs, assuming that STIs in need of inpatient care are less prone to diagnostic bias. This observation might further indicate a specific impact of bereavement on severer cases of STIs compared to other cases. Such a possibility was further supported by findings on acute salpingitis which is more exclusively diagnosed and treated in hospital-based inpatient or emergency outpatient care, and was therefore more completely ascertained using our register-based definition.

Since the Patient Register has limited information about the clinical characteristics of the STI diagnoses, whether or not the association of bereavement with STIs would differ for new infection, persistent infection, and recurrence of previous infection needs to be investigated. In a previous study, we showed that bereavement was associated with infection of human papilloma virus (HPV), especially high viral load infection and recurrent infection [13].

Another limitation of the present study concerns the definition of psychiatric disorders, especially among the bereaved women. Since we identified only psychiatric disorders diagnosed through inpatient or outpatient care, whether or not the increased risk of STIs noted among bereaved women without subsequent psychiatric disorders was due to milder psychiatric conditions or symptoms not yet attended by specialist care needs to be further examined.

Since we identified spouses of the study women through a common child, women in childless marriages would have been classified as unexposed even if they did lose their spouses. Similarly, since we had no information on whether a woman resided with the father of her children, the impact of loss of a spouse would differ for different women according to whether or not they were living with the father of her children. These would however most likely have led to an underestimate of the real impact of loss of a spouse on STIs.

Lastly, residual confounding might still exist, even though multiple potential confounders have been adjusted for, such as age, calendar periods, and parental education level.

The strengths of the present study include the large sample size, the nationwide study design, the long and complete follow-up, as well as the prospectively and independently collected information on the exposure and outcomes.

This study contributes to the growing body of knowledge on how stressful life events affect our health. While the importance of counseling grieving family members to be mindful of their health might seem obvious, there is still a lot to be done. Our study indicates that sexual risk behavior should especially be taken into account when counseling adolescents who have lost a family member, especially those youth who develop psychiatric disorders at the wake of their loss.

## Conclusions

Women who have lost a close relative are at increased risk of STIs. Bereaved women with subsequent psychiatric disorders may be at particularly high risk.

## Abbreviations

ATC, anatomical therapeutic chemical; CI, confidence interval; GHS, genital herpes simplex; HBV, hepatitis B; HIV, human immunodeficiency virus; HPV, human papillomavirus; ICD, international classification of diseases; IRR, incidence rate ratio; MGR, multi-generation register; STI, sexually transmitted infections

## Additional file

Additional file 1: Table S1. Swedish revisions of the international classification of diseases (ICD) codes for sexually transmitted infections. List of the ICD-9 and ICD-10 codes used to identify diagnosis of sexually transmitted infections. **Table S2.** Characteristics of women with or without bereavement. Description of parental educations levels, preexisting psychiatric disorders, and mean age, of the women in the cohort who had, or had not, experienced bereavement. **Table S3.** Hospital contact for sexually transmitted infections (STIs) after bereavement, among HPV vaccinated women. Analysis of STIs after bereavement, only including women who had received HPV vaccination. (DOCX 20 kb)
