# Supplementary material for: Sexually transmitted infections after bereavement – a population-based cohort study
Source: BMC Infect Dis. 2016 Aug 15;16:419. doi: 10.1186/s12879-016-1705-x (PMC4986385; doi:10.1186/s12879-016-1705-x)
Supplement: Additional file 1: Table S1. — Swedish revisions of the international classification of diseases (ICD) codes for sexually transmitted infections. List of the ICD-9 and ICD-10 codes used to identify diagnosis of sexually transmitted infections. Table S2. Characteristics of women with or without bereavement. Description of parental educations levels, preexisting psychiatric disorders, and mean age, of the women in the cohort who had, or had not, experienced bereavement. Table S3. Hospital contact for sexually transmitted infections (STIs) after bereavement, among HPV vaccinated women. Analysis of STIs after bereavement, only including women who had received HPV vaccination. (DOCX 20 kb) [file 12879_2016_1705_MOESM1_ESM.docx]

**Supplementary Table S1. Swedish revisions of the international classification of diseases (ICD) codes for sexual transmitted infections.**

|  | **ICD-9** | **ICD-10** |
| --- | --- | --- |
| Calendar period | 1987-1996 | 1997-2012 |
| Condyloma | 078B | A630 |
| Gonorrea | 098 | A54 |
| Chlamydia | 099B, 078J | A55-A56 |
| Syphilis | 091-097 | A51-A53 |
| Human immunodeficiency virus | 279K | B20-B24 |
| Anogenital herpes simplex | 054B | A60 |
| Acute salpingitis | 614A, 614C | N701, N709 |
| Acute hepatitis B | 070B | B16 |

**Supplementary Table S2. Characteristics of women with or without bereavement.**

|  | Women without bereavement | Women with bereavement |
| --- | --- | --- |
| Total number | 2,539,615 | 979,579 |
| Age at cohort entry – mean (SD) | 18.3±10.4 | 34.1±8.9 |
| Parental education level |  |  |
| High | 877,653 (34.6) | 138,114 (14.1) |
| Middle | 1,116,616 (44.0) | 307,959 (31.4) |
| Low | 503,965 (19.8) | 368,201 (37.6) |
| Unknown | 41,381 (1.6) | 165,305 (16.9) |
| Preexisting psychiatric disorders |  |  |
| Yes | 251,963 (9.9) | 45,204 (4.5) |
| No | 2,287,652 (90.1) | 934,375 (95.4) |

**Supplementary Table S3. Hospital contact for sexually transmitted infections (STIs) after bereavement, among HPV vaccinated women***

|  | **No Bereavement** | | | **Bereavement** | | |
| --- | --- | --- | --- | --- | --- | --- |
|  | **N** | **Crude IR**  (1000 PYs) | **IRR (95% CI)** | **N** | **Crude IR**  (1000 PYs) | **IRR (95% CI)** |
| **Ever Vaccinated** |  |  |  |  |  |  |
| Condyloma | 787 | 1.65 | 1.0 | 12 | 3.63 | 1.68 (0.89-2.83) |
| Any STI | 2165 | 4.53 | 1.0 | 23 | 6.95 | 1.16 (0.75-1.71) |
|  |  |  |  |  |  |  |
| **Fully Vaccinated** |  |  |  |  |  |  |
| Condyloma | 460 | 1.41 | 1.0 | 8 | 3.63 | 2.19 (1.00-4.11) |
| Any STI | 1510 | 4.63 | 1.0 | 16 | 7.25 | 1.32 (0.77-2.08) |

***The first three months following vaccination were excluded from the follow-up.**

The first hospital visit concerning an STI diagnosis during the unexposed follow-up and the first hospital visit concerning an STI diagnosis during the exposed follow-up were both counted in all analyses.

CI, confidence interval; IR, incidence rate; IRR, incidence rate ratio; N, number; PYs, person-years; STIs, sexual transmitted infections;

IRR was adjusted for attained age (10-18 and 19-44), calendar years (5-year group), and parental education levels (low/missing, medium, and high) as a proxy for socioeconomic status.
